# Case report: Recurrence of primary hepatic neuroendocrine tumors after resection of liver segments IV in 8 years follow-up

**DOI:** 10.3389/fmed.2024.1437650

**Published:** 2024-09-16

**Authors:** Chunli Li, Li Bian, Guangtao Fan, Yilong Huang, Jiang Li, Bo He

**Affiliations:** ^1^Department of Medical Imaging, The First Affiliated Hospital of Kunming Medical University, Kunming, China; ^2^Department of Pathology, First Affiliated Hospital of Kunming Medical University, Kunming, China; ^3^Medical Imaging Department, Yunnan Provincial Hospital of Traditional Chinese Medicine, Kunming, China; ^4^Hepatobiliary Surgery, The First Affiliated Hospital of Kunming Medical University, Kunming, China

**Keywords:** neuroendocrine tumors, liver, follow-up, recurrence, case report

## Abstract

**Background:**

Primary hepatic neuroendocrine tumors (PHNETs) are an utterly rare entity. The diagnosis of PHNETs could legitimize when an extrahepatic primary NET must always be excluded. PHNETs can achieve a high survival rate after complete surgical resection, however, most patients still have an 18% risk of recurrence within 5 years after surgery. In our case, the recurrence occurred 8 years after the first hepatectomy, which is relatively rare in the current literature. Therefore, rigorous postoperative follow-up is necessary for early detection and timely treatment of recurrent PHNETs.

**Case information:**

We report a case of PHNET in a 24-year-old previously healthy female patient who relapsed 8 years after hepatectomy. This case focuses on the importance of diagnosis of primary and recurrent PHNETS in young patients, rare pathological types, and post-operative follow-up.

**Conclusion:**

This case report detailed the rare pathological morphology and characteristic immunohistochemical markers in our case for PHNETS, which enhanced the new understanding of the diagnosis of this entity. In addition, we also highlighted the variable duration of recurrence after treatment of PHNETs. The 8-year recurrent period in our case suggests the importance of regular examination in patients with PHNETs by following the doctor’s instructions.

## Introduction

Neuroendocrine tumors (NETs) are rare heterogeneous tumors that arise from the neuroendocrine system (1). Although NETs can be found anywhere in the body, they primarily occur in the gastrointestinal tract, pancreatic, and bronchopulmonary systems (2). Neuroendocrine neoplasm originating from the liver is a rare tumor type, and most of them are metastases of neuroendocrine tumors from other sites. Whereas primary hepatic neuroendocrine tumors (PHNETs) are a less common subtype accounting for only 0.11% of primary hepatic tumors and 0.77% of NETs (3, 4). There are considered challenging to diagnose PHNETs owing to the radiological features with poor specificity. Therefore, the correct diagnosis of PHNETs mainly depends on histopathology, immunohistochemistry, and long-term follow-up to rule out an extrahepatic origin of NETs. In addition, the recurrent rate of five years for PHNETs is up to 18% despite complete surgical resection, so another purpose of long-term follow-up is to monitor tumor recurrence (5). Herein, we report a case of PHNET in a 24-year-old female patient who relapsed after 8 years of follow-up and was diagnosed through histopathology, immunohistochemistry, and follow-up outcomes. Our case report followed the CARE Guidelines.

## Case description

**Figure 1:**
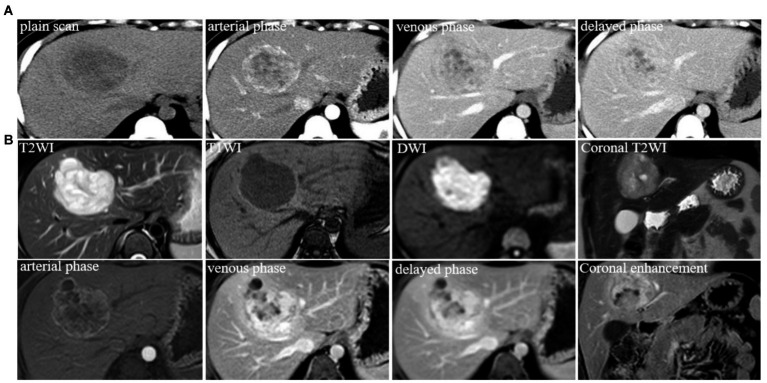
**(A)** Typical level and imaging findings of abdominal CT images for the lesion in the S4 segment of the liver; **(B)** abdominal MR typical images for the lesion in the S4 segment of the liver.

An asymptomatic 24-year-old woman visited our Department of Gastroenterology with a 2-month history of liver mass detected by a routine health check-up. Her previous surgical history was negative and physical examination was normal. The patient’s medical history revealed no significant abnormalities in the history of endocrine system diseases, infectious disease, allergic history, and no similar diseases or genetic diseases in the family. There was no abnormal finding in the laboratory examination including alpha-fetoprotein (AFP), carcinoembryonic antigen (CEA), Hepatitis B core antibody-IgM, and Hepatitis B virus pre-S1 antigen. Abdominal contrast-enhanced CT scan in June 2014 (Figure 1A) showed a large heterogeneous hypodense lesion with unclear boundaries in the S4 segment of the liver measured 7.1 × 6.1 cm. There was a marked inhomogeneous enhancement on the hepatic arterial phase and progressive enhancement on the venous phase and delayed phase, indicating a possible neoplastic lesion. MR images in June 2014 (Figure 1B) revealed a well-circumscribed, heterogeneous lobulated mass in the S4 segment of the liver with unevenly higher signal intensity on T2-weighted fast and diffusion images (b = 800), slightly lower signal intensity on delimitation and scar, and slightly lower signal on T1WI. The enhancement pattern was similar to CT, with compression of the adjacent middle hepatic vein. The initial diagnosis of the lesion was considered to be a benign neoplasm. Other examinations included a negative CT scan of the chest and gastrointestinal endoscopy in which chronic gastric and duodenal inflammation was found. Considering that the primary lesion was neoplastic, the clinician decided to perform surgical resection of liver segments IV.

**Figure 2:**
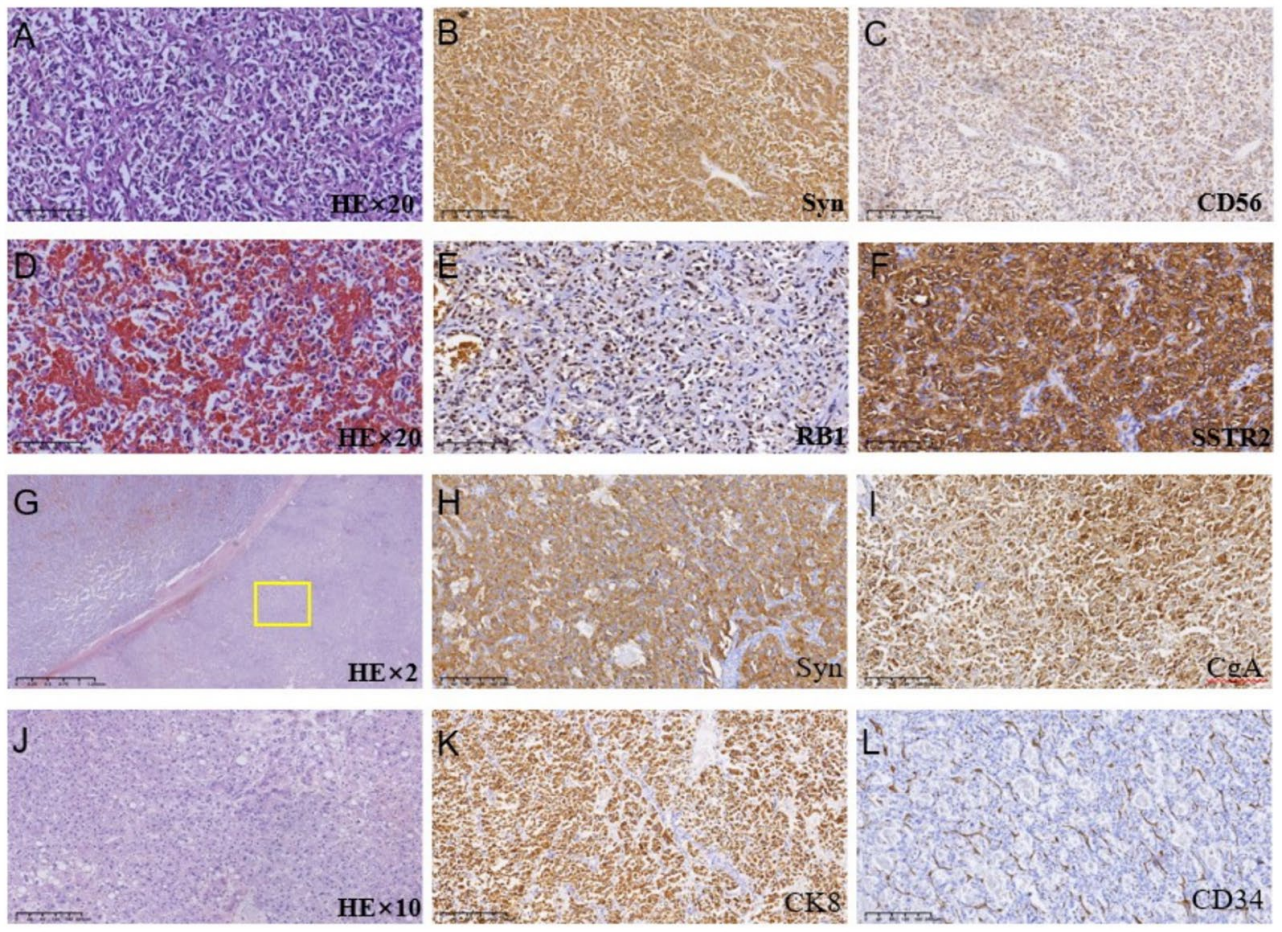
**(A–F)** Histologic images of tumor tissues by hematoxylin and immunohistological staining in the S4 segment of the liver; **(G–L)** Histologic images of tumor tissues by hematoxylin and immunohistological staining in the S7 segment of the liver.

**Figure 3:**
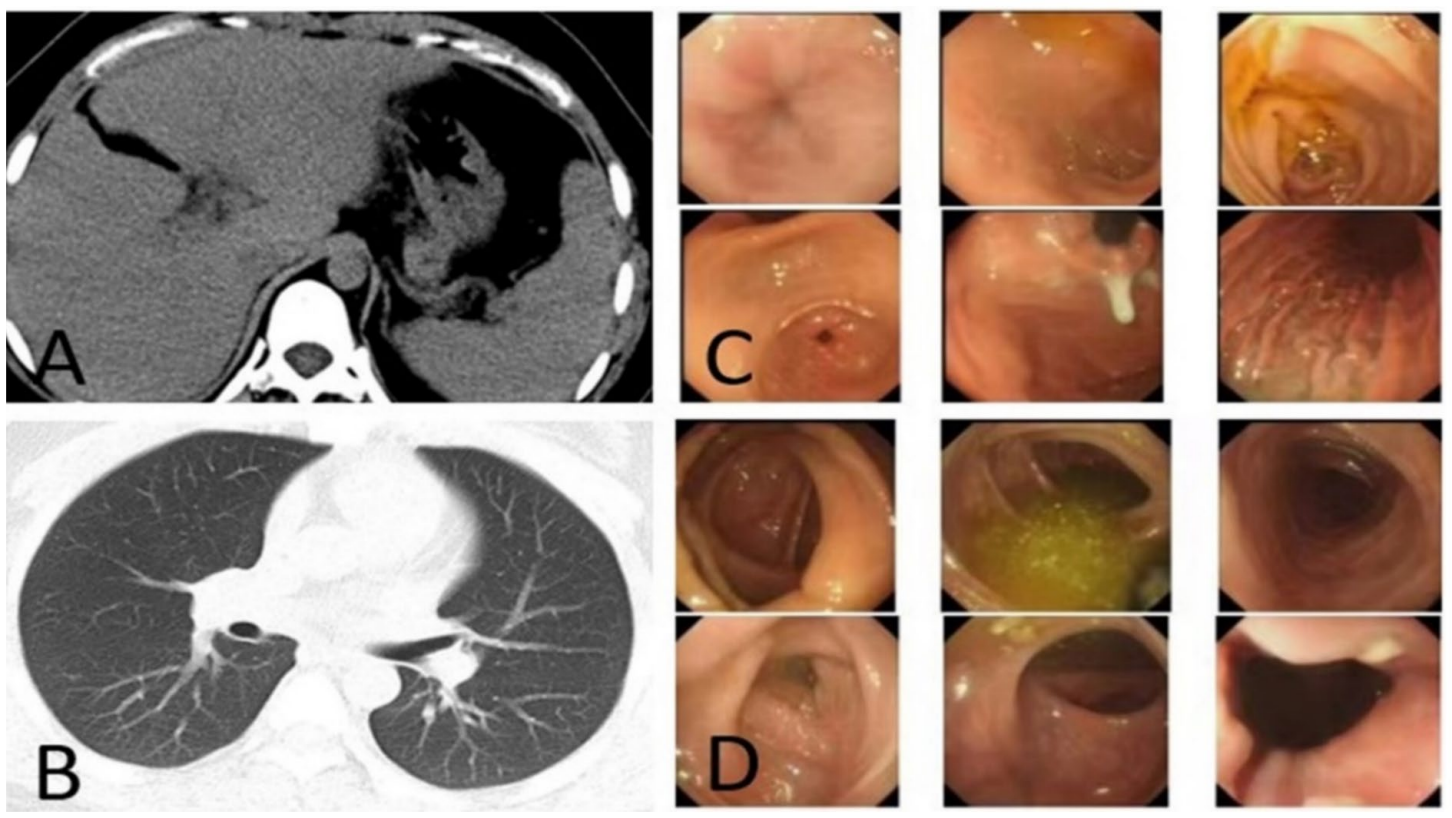
**(A)** CT image after resection the S4 segment of PHNET, **(B–D)** first post-operative negative images of chest CT, gastrointestinal endoscopy, and colonoscopy.

Because the patient is Rh blood type, after a month of blood preparation, she underwent resection of liver segments IV via the right upper abdomen. There was no active bleeding or bile leakage in the operative area. The patient’s vital signs were stable, the operative area pain gradually relieved and the incision recovered well. Histopathology of the resected lesion suggested a neuroendocrine tumor that subtype was hepatic goblet cell adenoid carcinoma. Immunohistological results were positive including chromgranin A (CgA), cluster of differentiation 56 (CD56), cytokeratin (CK)8/18, epithelial membrane antigen (EMA), and Villin (Figures 2A–F). Postoperative abdominal CT images showed that the density of hepatic parenchyma was homogeneous and no residual lesions were found (Figure 3A). The patient had an uneventful recovery after the procedure and underwent further investigation to rule out an extrahepatic origin of the NETs: gastrointestinal endoscopy, colonoscopy, and chest CT scan were negative (Figures 3B–D). And extra-hepatic primary NETs were not found during the two-year follow-up, therefore, the patient was finally diagnosed with PHNETs.

**Figure 4:**
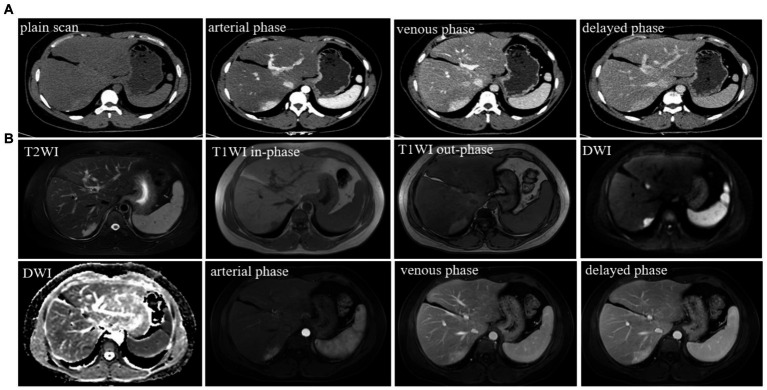
**(A)** Typical level and imaging findings of abdominal CT images for the recurrent lesion in the S7 segment of the liver; **(B)** abdominal MR typical images for the recurrent lesion in the S7 and S8 segment of the liver.

**Figure 5:**
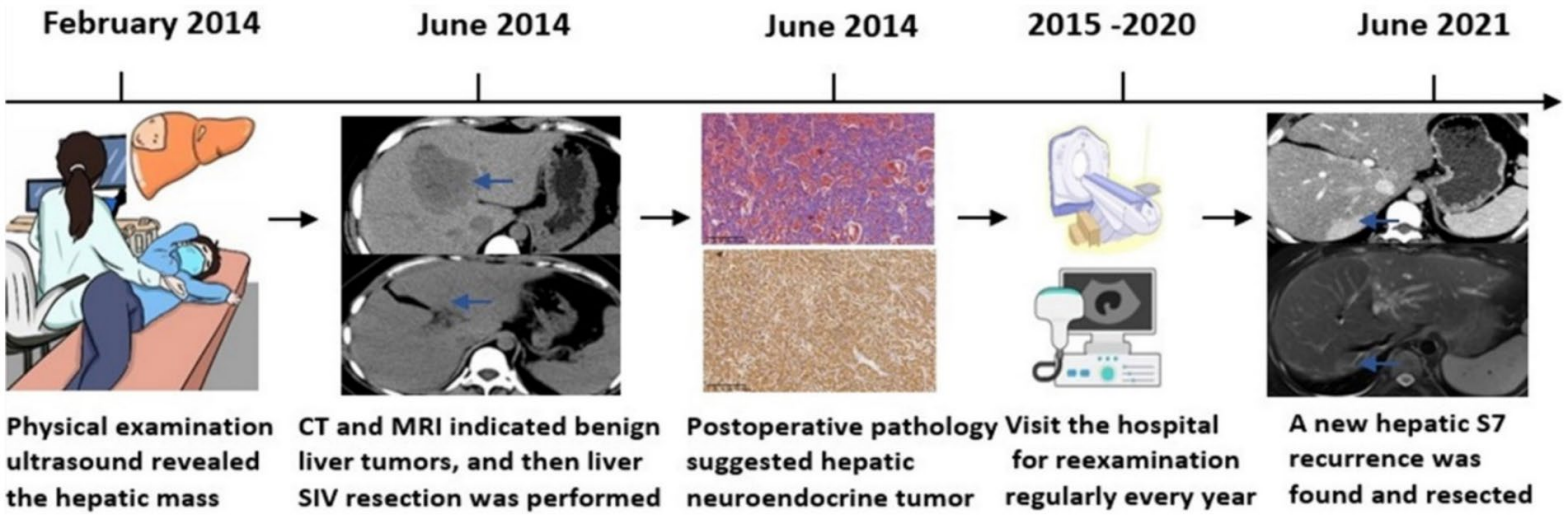
Graphical illustration of a timeline fashion the course of the PHNET in case.

The patient returned to the hospital regularly for re-examination every year to avoid oversight of recurrent foci (Table 1). Eight years later, the patient was admitted to the hospital complaining of abdominal pain. Laboratory tests including hepatitis markers, CA-125, CA199, AFP, and CEA remained negative. CT scanning in June 2021(Figure 4A) exhibited new small patches of high-density lesions in the S7 segment of the liver. The arterial and venous portal phase of dynamic enhancement CT showed even and obvious enhancement, and the degree of enhancement in the delayed phase declined but was still higher than that in the adjacent liver parenchyma. MR images in June 2021 (Figure 4B) slightly lower T1 and higher T2 signal foci were observed in the S7 and S8 segments of the liver presenting with a high signal intensity on DWI, and the enhancement pattern was consistent with CT. Finally, the radiologist and the hepatobiliary surgeon thought about the possibility of recurrence and decided to perform laparoscopic liver VII segmentectomy. Cholecystectomy was also performed because intraoperative exploration of the abdominal cavity found that the gallbladder adhered to the surrounding tissues. The immunohistochemical staining in the lesion cells was positive for CgA, Synaptophysin (Syn), CD 34, CK 8/18/19, EMA, and Villin (Figures 2G–L). The Ki-67 index was 5% suggestive of a grade 2 tumor. The medical history of the patient coupled with the immunohistochemical results and follow-up outcome supported the diagnosis of recurrence of PHNETs. Figure 5 shows the timeline for the occurrence and development course of the PHNET in our case.

**Table 1:** First Postoperative follow-up timeline of patients in our case.

Follow-up time	Review modality	Recurrent lesions
April 2015	Abdominal CT, MRI, and US, Chest DR	No
February 2016	Abdominal CT, MRI, and US, Chest DR	No
February 2017	Abdominal MRI and the US	No
May 2018	Abdominal MRI	No
July 2019	Abdominal and Chest CT	No
April 2020	Abdominal CT and Chest DR	No
June 2021	Abdominal CT, MRI, and US, Chest DR And CT	Yes
August 2022	Abdominal CT and MRI, Chest CT	No
February 2023	Abdominal CT and MRI, Chest CT	No
May 2024	Abdominal MRI, Chest CT	No

CT, Computed Tomography; MRI, Magnetic Resonance Imaging; US, Ultrasound; DR, Digital Radiography.

## Discussion

Since NETs originate from the neuroectodermal cells and do not routinely migrate to the liver, PHNTEs are a rare entity, which only comprises 0.3% of all NETs (6, 7). The origin of PHNEN remains unclear. Main theories have been proposed for the origin which arises from neuroendocrine cells in the epithelium of the intrahepatic biliary duct, or ectopic heterotopic pancreatic and adrenal tissue (8, 9). PHNETs most frequently occur in 40–50 years, the median age was 51.9 years, and there was no significant gender difference (10, 11). PHNETs are rare in patients younger than 40 years, which was first detected at the age of 24 in our case. Most patients with PHNTEs remain asymptomatic due to slow growth and lack of endocrine function (12). Some patients may present with non-specific symptoms such as abdominal pain, mass, weight loss, and jaundice (13). Only 6.8% of patients with PHNETs may present with carcinoid syndrome (11, 14).

CT imaging of PHNETs usually appears as low-density masses with significant enhancement in the arterial phase and declined enhancement in the portal or delayed phases (15). MRI of PHNETs typically exhibits rim-like enhancement on the arterial phase and continuous enhancement in the portal or delayed phase (16). The first imaging findings of our case were consistent with the above-mentioned CT imaging changes whereas recurrent lesions show marked homogeneous enhancement in the arterial and portal phases, with reduced enhancement in the delayed phase. The imaging findings of this case once again confirmed that radiological findings of PHNETs were non-specific. Due to the lack of sensitive tumor markers and specific imaging findings of NTEs, Pathological results particularly immunohistochemical results are required for the definitive diagnosis of NETs (17, 18).

Pathological morphology of PHNETs included insular, nested, trabecular, or mixed pattern of cell growth pattern (11). The pathological type of our case was goblet cell adenoid carcinoid, which is extremely rare in PHNETs and is considered a subgroup of mixed neuroendocrine neoplasmsand adenocarcinomas (19). The spectrum of neuroendocrine tumors encompasses a variety of entities arising in different organs, including those that are considered intermediate and controversial and are grouped under terms such as “goblet cell carcinoid” (20). Neuroendocrine tumors presenting as Goblet cell carcinoids occur almost exclusively in the appendix reported in the literature to date (21). Only a few cases present with extraappendiceal locations such as the stomach, ileum, cecum, ascending colon, hepatic exure, sigmoid, and rectum (22). Goblet cell carcinoids primarily affect the appendix, although they have been reported in other locations within the gastrointestinal and biliary tract. However, goblet cell adenoid carcinoma occurring in the liver is extremely rare and has not been reported in the literature so far. The main immunohistochemical markers of PHNETs include chromogranin A (CgA), neuro-specific enolase (NSE), synaptophysin (Syn), and CD56 (12, 17, 23). CgA, NSE, and SYP were found in both primary and recurrent lesions in our case. However, immunohistochemical markers detected in our case and not reported in other hepatic neuroendocrine neoplasms include villin. Villin is a protein of the brush border of epithelial cells, which is a main immunohistochemical marker in colorectal and gastrointestinal neoplasms. Negative expression of Viilin was found in histopathological examination of a pulmonary metastatic tumor from uterine sarcoma in a rare case (24). Frequent and high-level villin can be found immunohistochemically in 22–41% of neuroendocrine neoplasms (25). Gong et al. proposed that the combination of villin and CDX-2 could be utilized for determining the primary origin of metastatic cancer (26). In addition, data from Arango et al. confirmed a loss of villin expression was associated with advanced tumor stage, nodal metastasis, and microsatellite instability of colorectal cancer (27). Therefore, the value of villin immunohistochemistry for the identification of hepatic neuroendocrine neoplasms is worthy of further exploration. According to the latest NETs guidelines, the classification of PHNETs depends primarily on Ki-67, which is a vital marker of tumor proliferation. Ki-67 index can divide NETs into low and intermediate-grade neuroendocrine neoplasms (grade 1 and 2), and high-grade neuroendocrine carcinomas (grade 3). Grade1-3 tumors are often characterized by Ki-67 index<3%, between 3–20%, and > 20% (28). Although the diagnosis and grading of hepatic neuroendocrine tumors can be achieved by pathological features and the ki-67 index, it is difficult to differentiate primary hepatic neuroendocrine carcinomas from metastases using pathological evidence alone. Therefore, We need a continuum including testing before surgery, examination during surgery, pathology, and follow-ups after surgery to rule out extra-hepatic primary lesions for accurate diagnosis of PHNETs.

There are no guidelines for the treatment of PHNETs, but early detection and complete resection of the lesions are the preferred safe and effective treatment options. Literature reports that the 5-year survival rate after surgical resection is 74–78% (14, 29). Other treatment methods include transarterial chemo-embolization (TACE), chemotherapy, radiotherapy, somatostatin analogs, and conservative therapy (30–32). Although surgical resection of PHNETs has a high survival rate, most patients still have an 18% risk of recurrence within 5 years after surgery (33). Recurrence time was variable among patients; from a few months to a few years in the literature (Table 2). In our case, the recurrence occurred 8 years after the first hepatectomy, which is relatively rare in the current literature. Rigorous postoperative follow-up is necessary for early detection and timely treatment of PHNETs. Local recurrence can be detected early in our case due to the strictly regular follow-up visits (Table 1). Therefore, appropriate treatment of the patient was carried out in time so that the patient keeps symptom-free and has a good health status so far. Ki-67 protein is an important predictor of tumor recurrence (34). A Ki-67 index <2% has been shown to have a better prognosis in patients with malignant pancreatic NETs (35). Therefore, clinicians should pay attention to the role of the Ki-67 index in patients with PHNETs.

**Table 2:** The summary of clinical characteristics, immunohistochemical findings, and status of recurrent patients in current literature.

Author/year	No.	Sex/age	Treatment methods	Immunohistochemical findings	Timing of recurrence (mo)	Recurrence treatment
Jung et al. (2023) (17)	1	F/37	LH + BDR + PVT	Syn(+), CgA(+)	13	CTX
	2	F/64	RH	Syn(+)	47	RTX
	3	M/62	CBS	Syn(+), CgA(+)	1	TACE
	4	M/50	LTS	Syn(+), CgA(+), CD56(+)	2	None
	5	F/48	LTS	Syn(+)	23	TACE
	6	M/26	CBS	Syn(+), CgA(+)	5	TACE
Tang et al. (2023) (36)	7	F/72	Partial S7 hepatectomy	Syn(+), CD56(+)	48	N/D
Nishimori et al. (2005) (37)	8	M/70	LH	Syn(+), CgA(+)	10/156	hepatectomy
Iimuro et al. (2002) (38)	9	M/71	hepatectomy	CgA(+), NSE(+)	67	N/D
Abdel et al. (2006) (39)	10	F/32	LH	N/D	120	hepatectomy
Shah et al. (2019) (12)	11	M/35	LH+ wedge resection of the right lobe nodule	Syn(+), CD56(+), NSE(+)	N/D	hepatectomy and LAR
	12	M/56	RH	NET(+), CK7(+), Syn(+), CgA(+), CD56(+)	N/D	LAR
Zhang et al. (2008) (40)	13	F/61	RH + CBS	Syn(+), CgA(+), NSE(+)	48	N/D
	14	M/35	LH + S1	Syn(+), CgA(+), NSE(+), CK(+)	79	N/D
	15	F /47	LH	Syn(+), CgA(+), NSE(+), CK(+)	41	N/D
Clayton et al. (2003) (5)	16	M/ 41	CBS + S1	CgA(+), NSE(+), serotonin(+)	9/72	hepatectomy
Hwang et al. (2008) (34)	17	F/ 37	LH + BDR + PVT	Syn(+), CgA(+)	13	CTX
	18	M/62	CBS	Syn(+), CgA(+)	1	TACE
	19	M/50	LTS	Syn(+), CgA(+), C D56(+)	2	None

LH, left hepatectomy; BDR, bile duct resection; PVT, portal vein thrombectomy; CTX, chemotherapy; CBS, central bisectionectomy; LTS, left trisectionectomy; LMS, left medial sectionectomy; S1, caudate lobe resection; TACE: ftransarterial chemo-embolization.

## Conclusion

PHNETs are a rarity that can only legitimize the diagnosis of PHNETs when an extrahepatic primary NET must always be excluded. Although most patients are middle-aged and old, the possibility of PHNETs in young people should not be missed. The diagnosis of PHNETs remains challenging. Histopathological examination and immunohistochemical results can provide an important standard for the diagnosis of PHNETs. The enlightenment of this case is that patients with PHNETs should follow the doctor’s instructions to the hospital regularly for reexamination Although PHNETs are benign, they still have a high recurrence rate after surgery, and high-grade PHNETs have a high risk of malignancy. Therefore, Regular visits to the hospital are beneficial for early accurate diagnosis and treatment of recurrent PHNETs. However, most patients exhibit poor adherence owing to individual factors and lack appropriate guidelines for regular interval reviews, making it challenging to carry out the scheduled reexamination plan. The case provides valuable insights into the significance of follow-up. Early treatment after recurrence with curative intent can prolong survival and improve the patient’s quality of life.

## Data Availability

The original contributions presented in the study are included in the article/Supplementary material, further inquiries can be directed to the corresponding author.
