# Supplementary figures and images for: Case report: Recurrence of primary hepatic neuroendocrine tumors after resection of liver segments IV in 8 years follow-up
Source: Front Med (Lausanne). 2024 Sep 16;11:1437650. doi: 10.3389/fmed.2024.1437650 (PMC11439660; doi:10.3389/fmed.2024.1437650)

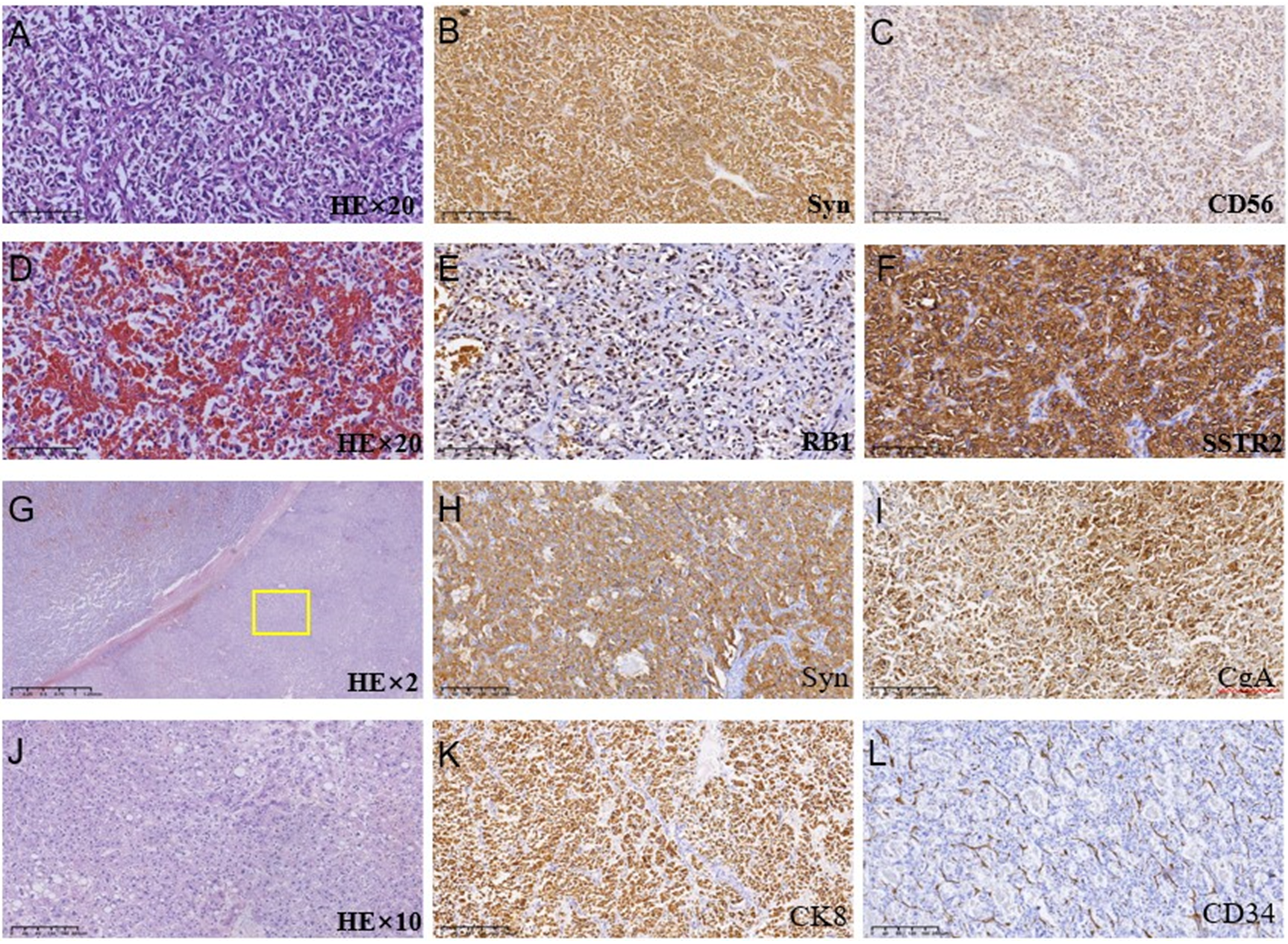

Supplement: Supplementary file 1 [file Image_1.TIF]

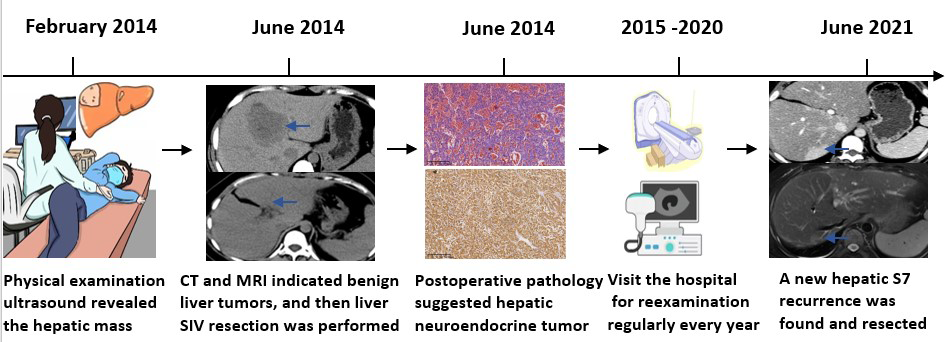

Supplement: Supplementary file 2 [file Image_2.TIF]
